# Supplementary material for: Label-free Evaluation of Myocardial Infarct in Surgically Excised Ventricular Myocardium by Raman Spectroscopy
Source: Sci Rep. 2018 Oct 2;8:14671. doi: 10.1038/s41598-018-33025-6 (PMC6168494; doi:10.1038/s41598-018-33025-6)
Supplement: Supplementary file 2 — Supplementary information [file 41598_2018_33025_MOESM2_ESM.pdf]

Label-free evaluation of myocardial infarct in surgically excised ventricular myocardium by Raman spectroscopy

Tsunehisa Yamamoto[1,2,¶], Takeo Minamikawa[1,3,4,¶,*], Yoshinori Harada[1], Yoshihisa Yamaoka[5], Hideo Tanaka[1], Hitoshi Yaku[2], and Tetsuro Takamatsu[1,6,*]

[1]Department of Pathology and Cell Regulation, Graduate School of Medical Science, Kyoto Prefectural University of Medicine, 465 Kajii-cho Hirokoji Kawaramachi, Kamigyo-ku, Kyoto 602-8566, Japan

[2]Department of Cardiovascular Surgery, Graduate School of Medical Science, Kyoto Prefectural University of Medicine, 465 Kajii-cho Hirokoji Kawaramachi, Kamigyo-ku, Kyoto 602-8566, Japan

[3]Department of Mechanical Science, Division of Science and Technology, Graduate School of Technology, Industrial and Social Sciences, Tokushima University, 2-1 Minami-Josanjima, Tokushima, Tokushima 770-8506, Japan

[4]PRESTO, Japan Science and Technology Agency (JST), 2-1 Minami-Josanjima, Tokushima, Tokushima 770-8506, Japan

[5]Department of Advanced Technology Fusion, Graduate School of Science and Engineering, Saga University, 1 Honjo, Saga, 840-8502, Japan

[6]Department of Medical Photonics, Kyoto Prefectural University of Medicine, 465 Kajii-cho Hirokoji Kawaramachi, Kamigyo-ku, Kyoto 602-8566, Japan

[¶]Co-first author (equal contribution)

[*]Correspondence and requests for materials should be addressed to Takeo Minamikawa (minamikawa.takeo@tokushima-u.ac.jp) or Tetsuro Takamatsu (ttakam@koto.kpu-m.ac.jp).

Supplementary Figures

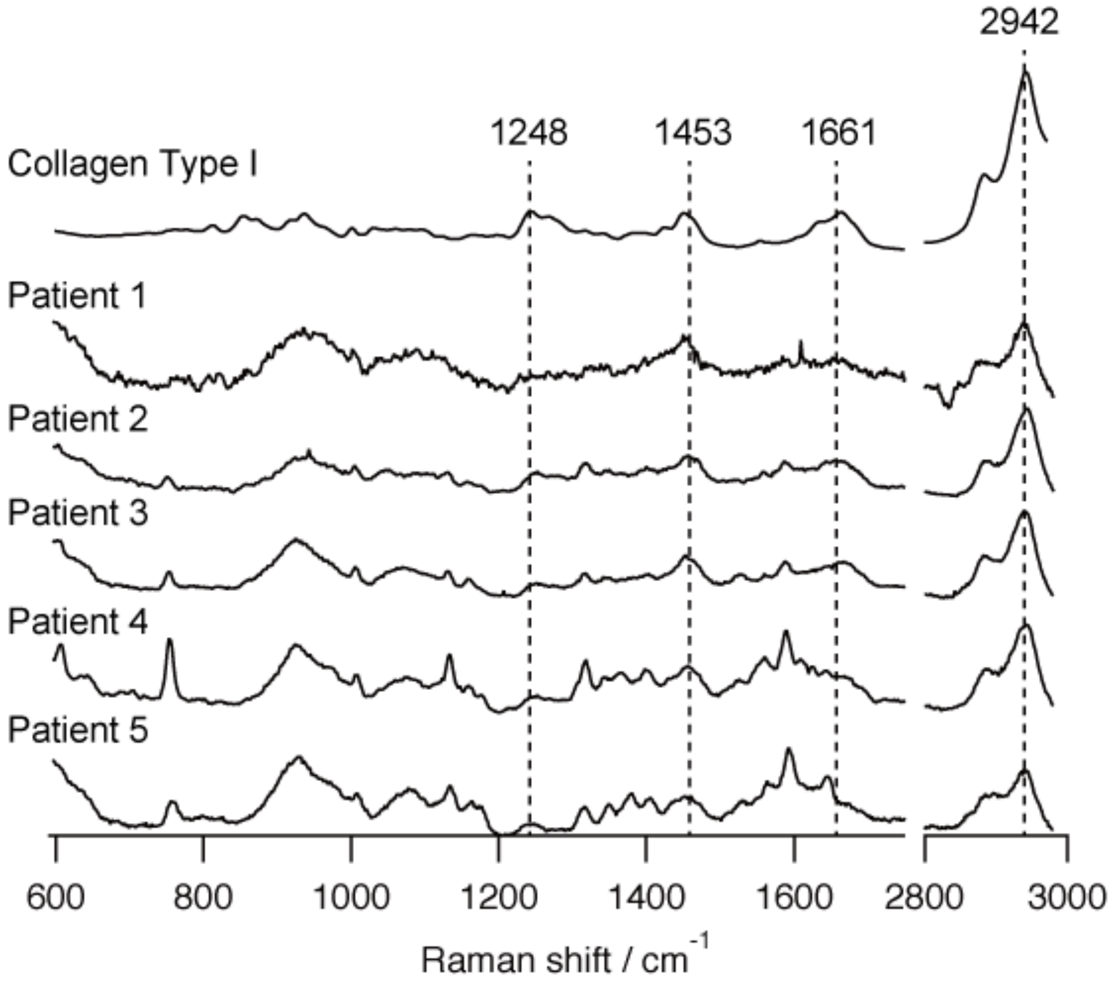

Supplementary Figure 1. Normalized Raman spectra of pure chemical of collagen and human infarcted myocardium.

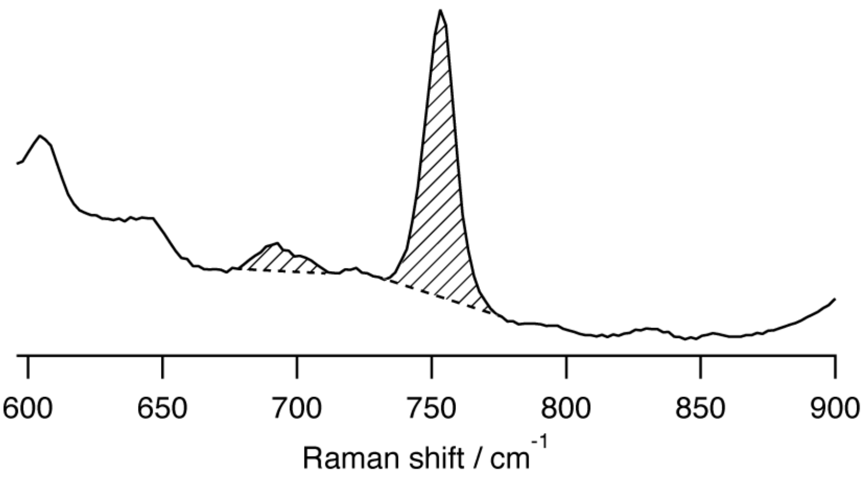

Supplementary Figure 2. Definition of area intensity. Shaded regions indicate area intensities of 687 and 755 cm^{-1}.

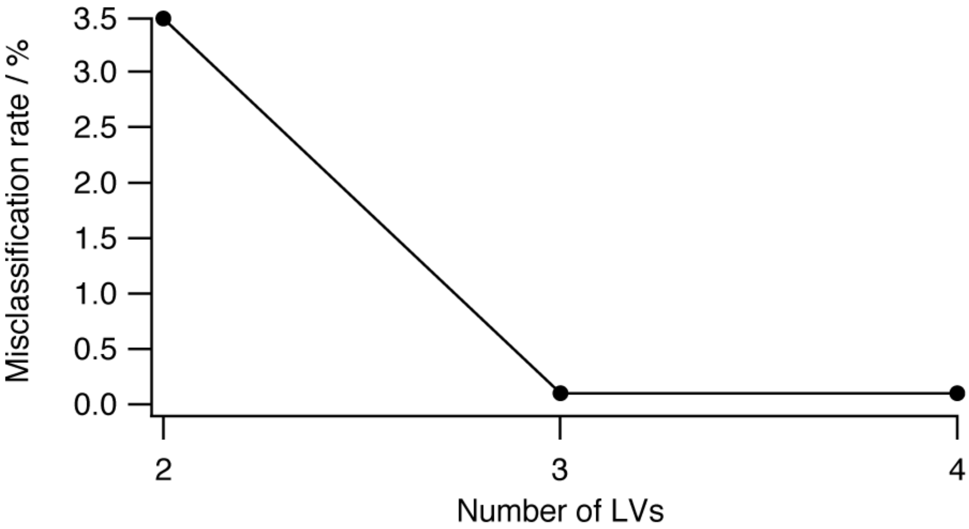

Supplementary Figure 3. Misclassification rate depending on number of LVs. First minimum misclassification rate was at the number of three of LVs.

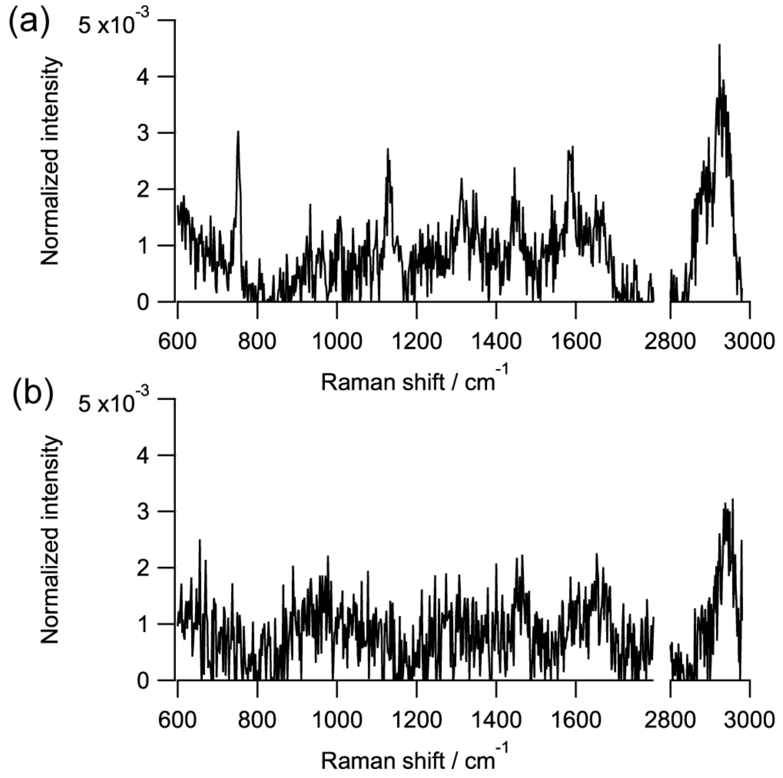

Supplementary Figure 4. Typical normalized Raman spectra obtained under the condition of 10-s exposure of 532-nm excitation at about 250 μW/μm^2. (a) Typical normalized Raman spectrum of a infarcted region. (b) Typical normalized Raman spectrum of non-infarcted region.

Supplementary Table 1. Cross-validated detection power of non-infarcted and infarcted myocardium of patient 1, in which the prediction model was constructed with the data of the other patients. Detection accuracy for the prediction was 96.80%.

	Histology	
	Non-infarcted	Infarcted
Non-infarcted	1000	64
Infarcted	0	936
Sensitivity	100%	93.60%

Supplementary Table 2. Cross-validated detection power of non-infarcted and infarcted myocardium of patient 2, in which the prediction model was constructed with the data of the other patients. Detection accuracy for the prediction was 100%.

	Histology	
	Non-infarcted	Infarcted
Non-infarcted	1000	0
Infarcted	0	1000
Sensitivity	100%	100%

Supplementary Table 3. Cross-validated detection power of non-infarcted and infarcted myocardium of patient 3, in which the prediction model was constructed with the data of the other patients. Detection accuracy for the prediction was 99.45%.

	Histology	
	Non-infarcted	Infarcted
Non-infarcted	989	0
Infarcted	11	1000
Sensitivity	98.90%	100%

Supplementary Table 4. Cross-validated detection power of non-infarcted and infarcted myocardium of patient 4, in which the prediction model was constructed with the data of the other patients. Detection accuracy for the prediction was 99.70%.

	Histology	
	Non-infarcted	Infarcted
Non-infarcted	994	0
Infarcted	6	1000
Sensitivity	98.90%	100%

Supplementary Table 5. Cross-validated detection power of non-infarcted and infarcted myocardium of patient 5, in which the prediction model was constructed with the data of the other patients. Detection accuracy for the prediction was 100%.

	Histology	
	Non-infarcted	Infarcted
Non-infarcted	1000	0
Infarcted	0	1000
Sensitivity	100%	100%

Supplementary Table 6. Cross-validated detection power of non-infarcted and infarcted myocardium of all patient, in which the prediction of tissue species of a patient was performed with the prediction model constructed with the data of the other patients. Detection accuracy for the prediction was 99.19%.

	Histology	
	Non-infarcted	Infarcted
Non-infarcted	4983	64
Infarcted	17	4936
Sensitivity	99.66%	98.72%

Supplementary Movie

Supplementary Movie 1. Score plot of LV1, LV2, and LV3. Triangle, non-infarcted cardiac tissue; circle, infarcted cardiac tissue; red, patient 1; green, patient 2; blue, patient 3; yellow, patient 4; and purple, patient 5.
